# The Snow Must Go On: Ground Ice Encasement, Snow Compaction and Absence of Snow Differently Cause Soil Hypoxia, CO_2_ Accumulation and Tree Seedling Damage in Boreal Forest

**DOI:** 10.1371/journal.pone.0156620

**Published:** 2016-06-02

**Authors:** Françoise Martz, Jaana Vuosku, Anu Ovaskainen, Sari Stark, Pasi Rautio

**Affiliations:** 1 Natural Resources Institute Finland (Luke), Rovaniemi, Finland; 2 Arctic Centre, University of Lapland, Rovaniemi, Finland; WSL Institute for Snow and Avalanche Research SLF, SWITZERLAND

## Abstract

At high latitudes, the climate has warmed at twice the rate of the global average with most changes observed in autumn, winter and spring. Increasing winter temperatures and wide temperature fluctuations are leading to more frequent rain-on-snow events and freeze-thaw cycles causing snow compaction and formation of ice layers in the snowpack, thus creating ice encasement (IE). By decreasing the snowpack insulation capacity and restricting soil-atmosphere gas exchange, modification of the snow properties may lead to colder soil but also to hypoxia and accumulation of trace gases in the subnivean environment. To test the effects of these overwintering conditions changes on plant winter survival and growth, we established a snow manipulation experiment in a coniferous forest in Northern Finland with Norway spruce and Scots pine seedlings. In addition to ambient conditions and prevention of IE, we applied three snow manipulation levels: IE created by artificial rain-on-snow events, snow compaction and complete snow removal. Snow removal led to deeper soil frost during winter, but no clear effect of IE or snow compaction done in early winter was observed on soil temperature. Hypoxia and accumulation of CO_2_ were highest in the IE plots but, more importantly, the duration of CO_2_ concentration above 5% was 17 days in IE plots compared to 0 days in ambient plots. IE was the most damaging winter condition for both species, decreasing the proportion of healthy seedlings by 47% for spruce and 76% for pine compared to ambient conditions. Seedlings in all three treatments tended to grow less than seedlings in ambient conditions but only IE had a significant effect on spruce growth. Our results demonstrate a negative impact of winter climate change on boreal forest regeneration and productivity. Changing snow conditions may thus partially mitigate the positive effect of increasing growing season temperatures on boreal forest productivity.

## Introduction

At high latitudes, the climate has warmed at twice the rate of the global average, and as a consequence, northern forest plants are experiencing environmental conditions that are highly variable compared to in the past. The increase in temperature does not concern only the growing season. In fact, temperatures during the winter months in Finland have increased even more than those during the growing season [1]. Increasing winter temperatures have several impacts on the properties of snowpack, especially due to warm spells and rain-on-snow events. Data has shown that the frequency of rain-on-snow events has increased in Arctic regions during the last 30 years [2] and temperatures are projected to increase even further in the future [3]. In Finland, the duration and thickness of snow cover are expected to decrease and models predict lower snow water equivalent and higher ice content in the snowpack by the end of the 21st century [4]. Although boreal winter conditions are changing rapidly, surprisingly few experimental investigations exist on the effects of altered snow conditions on boreal forest plants or their winter survival.

Snowpack properties are of special importance for tree seedlings and understory forest plants because they are covered by snow for the whole winter. Numerous cellular processes are suppressed in overwintering plants upon entry into dormancy in the autumn, however maintenance of some metabolism is necessary to sustain defense and rescue processes in dormant tissues [5]. Similar to aboveground parts, maintenance respiration in roots may continue throughout winter even at near-freezing temperatures, even though root growth is considered to be limited below about 4°C [6, 7]. Changes in the snowpack alter the environmental overwintering conditions of conifer seedlings in two important ways. Firstly, a thinner or a denser snowpack has a lower insulation capacity, which may considerably decrease subnivean temperatures during the winter. The insulating snowpack decouples soil temperatures from air temperatures, allowing soil processes generally to continue throughout the winter and providing frost protection to soil microorganisms, roots and fauna. Secondly, increasing frequency of rain-on-snow events and higher winter temperature fluctuations lead to repeated freeze-thaw events that can create ground ice encasement (IE). The consequences of IE have so far been investigated mostly in northern agricultural landscapes, where it is known to cause injuries and kill overwintering cereals [8] and perennial grasses [9, 10].

Ice layers in the snow or on the ground surface restrict soil-atmosphere gas exchange, which leads to the development of hypoxia and accumulation of trace gases in the subnivean environment [11, 12]. Return to normoxia after long exposure to hypoxia also represents a major oxidative stress that can be fatal to plants [11]. Although the effects of the above changes in the snowpack on soil temperatures have been quite well studied, less is known about their influence on the amount of oxygen in soil, and the potential consequences these might have on overwintering conifer seedlings. Given that the snow-covered period can last more than half a year in boreal ecosystems, this creates a major gap in the understanding of how climate warming will affect boreal ecosystem functioning.

We studied the effects of changing snow conditions on soil microclimate and soil properties as well as on the survival and growth of conifer seedlings in a northern boreal coniferous forest. The study focused on Norway spruce and Scots pine as they are the two most common and economically important tree species in Fennoscandian boreal forests. We subjected experimental plots to several different snow manipulation treatments, which each represented different scenarios of how winter climate change may affect snow conditions and snowpack properties. By decreasing soil temperatures or creating hypoxia during the winter, we hypothesized that snow cover removal, snow compaction and IE will increase winter mortality and decrease the regrowth of young conifer seedlings during the following summer, thus inducing negative effects on both the productivity and the regeneration of forest trees.

## Material and Methods

### Study site and snow manipulation treatments

We established a snow manipulation experiment following a randomized block design with 10 blocks in a Scots pine forest (xeric- sub-xeric site) near the Arctic Circle close to the city of Rovaniemi (Tavivaara, 66°25 '35"N 25°41'42"E). The land used for the field experiment described below is owned by the Rovaniemi parish who allowed us to run the experiment. The field study did not involve endangered or protected species. The blocks were positioned on an area of approximately three hectares and organized in such a way that within each block the understrorey vegetation and tree layer were as homogenous as possible. The size of the blocks was typically around 100–200 m^2^ to allow treatment plots (see below) within each block to be positioned far enough from each other. Each block was fenced to prevent big herbivores to enter the treatment plots. The forest soil at the experimental site is acidic with a soil pH of 3.76 ± 0.03 (mean ± SE of three sample collections over the growing season, n = 30). The concentration of NH_4_-N, NO_3_-N and extractable organic nitrogen (N) in the humus layer was 5.7 ± 0.9, 1.2 ± 0.1 and 74.8 ± 7.3 mg kg DW^-1^ (n = 30), respectively, in ambient (AMB) plots during the 2014 growing season. Ground layer vegetation is dominated by red-stemmed feather moss (*Pleurozium schreberi*) and reindeer lichens (*Cladonia* spp.) and field layer vegetation is dominated by evergreen and deciduous dwarf shrubs (*Vaccinium vitis-idaea*, *V*. *myrtillus*, *V*. *uliginosum* and *Empetrum nigrum*). Local temperature, precipitation and snow depth values were recorded at the permanent weather station of the Finnish Meteorological Institute at Rovaniemi, Apukka, located 15km north of the field site (World Meteorological Organisation Station 02813, 66°34’N 26°00’E 106 m a.s.l.). The long-term (1981–2010) mean annual temperature in Apukka is 0.4°C with a mean precipitation of 556 mm [13] (S1A Fig). December, January and February are the coldest months of the year. The first permanent snow typically appears in mid-November, the snowpack is the highest in April with 60 cm (in open areas) and the average ending date of permanent snow cover is in early May. Forests typically have less snow cover than open areas [14]. To estimate the local effect of tree canopies and stand structure on the snow cover at the experimental site, we measured the depth of intact snow cover at our field site in winters 2015 and 2016 (19 March and 14 April 2015, 19 February, 31 March and 4 April 2016). Comparison with the data in the WMO station of Apukka showed that as an average, the snow cover in our filed site was about half that in the open area in Apukka (54.0 ± 8.7%, n = 5). Recording of air temperature (2-m height) showed that during November 2013–July 2014 the temperature was 0.61°C colder at our forest site than in the closest WMO station in Apukka during the same period.

**Fig 1:**
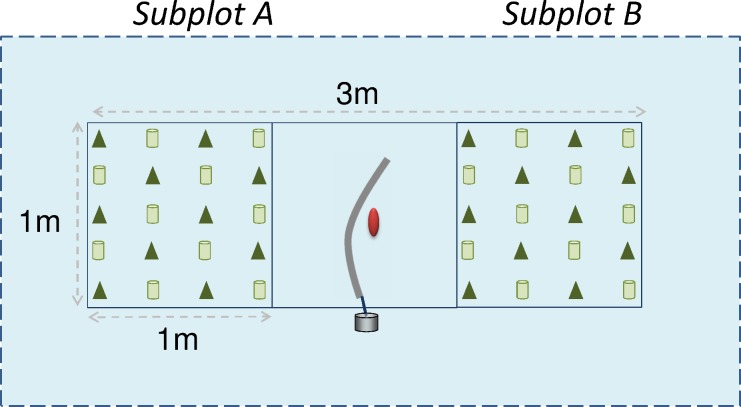
Plot design. Red dot = temperature logger; tube = air-collecting silicon tube inserted in humus layer; triangle = Norway spruce seedling; can = Scots pine seedlings; blue area = treated area.

Five study plots with similar exposure, hydrological status and vegetation were selected within each of the 10 blocks and randomly assigned to the following treatments: 1) ambient (AMB) with no treatment, 2) prevention of IE formation during winter (NoICE, plastic shelters were placed on a wood structure to cover the plot in case of a naturally occurring rain-on-snow event), 3) induction of IE (IE, artificially created by snow watering), 4) no snow cover (NoSNOW: roofs and low walls made of translucent white plastic (thickness 0.2mm) were positioned over the plots to prevent natural snow fall or drifted snow) and 5) compaction of snow cover (COMP: snow compaction by hand to reduce the insulating capacity of snow). Plots were located below tree canopies that were as similar as possible. Plots (1 m x 3 m) were divided into three sections: two subplots (A and B, both 1 m x 1 m) separated by a buffer zone (1 m x 1 m; Fig 1). The treated area extended 0.5 m beyond the edges of the plot. A subplots were used to take soil and biochemical samples from the seedlings in the beginning of the 2014 growing season for further analyses. B subplots were used to record seedling growth and health during the 2014 growing season (this study).

Snow watering was done by applying cold tap water using plastic watering cans over the extended plot area (in total 8 m^2^, Fig 1). Snow watering was conducted three times over the winter. The first watering was performed on December 13, 2013 (3.75 l m^-2^) but a warm spell prevented ice formation and thawed most of snow and ice away. A second watering was performed on December 17, 2013 (7.5 l m^-2^). A third watering was done on January 9, 2014 (7.5 l m^-2^). Watering corresponded to a total of 18.75 mm of rain. Snow compaction was applied by hand on December 13, 2013, January 9 and January 29, 2014 with care not to damage the seedlings. Our aim being to mimic mainly early winter warming, the snow manipulation treatments were done not later than January and natural snow was covering the IE and COMP plots after snow manipulation. Plastic shelters on NoICE plots were put up several times over the winter during natural rain-on-snow events: November 14–20, December 11–12, December 25, 2013, January 3, March 7–8 and 13–14, 2014. The plastic roofs over the NoSNOW plots were set in place on October 24, 2013 and removed on April 25, 2014. To limit any possible greenhouse effect, the open sides were widened by cutting the upper part of plastic walls in early March when it was considered unlikely that wind would blow any snow into the treated area. The white color of the translucent plastic roofs allowed maintaining the albedo of the NoSNOW plots similar to the snow-covered plots until the end of April. Temperature loggers were installed 30 cm above the ground on December 17, 2013 in AMB and NoSNOW plots in three blocks to check for any effect of the plastic roofs on air temperature. Temperature was recorded every 10 min; the difference between AMB and NoSNOW treatments was computed for each time point and averaged per hour. The results showed that the presence of the plastic roofs increased the mean daily air temperature on average by 0.144 ± 0.004°C (n = 18008 measurements).

Ambient air (2-m height), ground surface and humus (2-cm depth) temperature were recorded at 1.5-h intervals using temperature loggers (EL-USB-1, Lascar electronics) installed in every plot of three randomly selected blocks. Soil frost depth was followed by installing frost tubes filled with methylene blue solution in silicone tubes (2 m long) placed into tight PVC guide pipes inserted vertically into 2-m deep holes dug in the soil [15]. The soil frost probes were placed in the center of the buffer zone, close to the air sampling tube and temperature loggers (Fig 1). Frost depths were recorded on a weekly basis from early December to mid-June.

Pictures from all subplots A (n = 50) were taken on April 24, 2014 with a digital camera (Sony α-300). The perspective of the planted area was corrected in Photoshop (CS3 version 10.0.1). Snow covered-areas were manually selected in ImageJ1.49B and the proportion of snow cover was calculated as the ratio of snow-covered pixels to the total pixel number.

### Soil gas sampling and analysis

O_2_ and CO_2_ concentrations in soil were measured regularly over the winter from December 4, 2013 to May 14, 2014 when soil was fully thawed. The method of soil gas diffusion into silicon tubes was used [16]. In autumn 2013, silicon tubes (internal diameter 1.0 cm, wall thickness 0.3 cm, length 100 cm, V = 78.5 cm^3^) closed on both ends with silicon stoppers were installed horizontally in the middle of the buffer zone at a 2 cm depth in the humus layer. Bended stainless steel tubes (internal diameter 1 mm) inserted into a silicon stopper in one side of the silicon tubing allowed connecting it to the surface. The steel tubes were equipped with a three-way stopcock to allow gas sampling 1 m above the soil surface and outside of the plot to avoid trampling when sampling. Gas samples of 30 ml were taken with polypropolene syringes equipped with a three-way stopcock, stored at -20°C for a maximum of 24 h and analyzed by gas chromatography (Agilent 6890N) using a ShinCarbon ST 100/120 mesh 2 m x 1mm ID micropacked column (Restek). The effect of storage at -20°C in a polypropolene syringe on O_2_ and CO_2_ concentrations was tested using standard gas mixtures (N_2_, CO_2_) or soil samples with high CO_2_ concentrations but no effect was found for storage up to 48 h (data not shown). Samples were injected with a 1-ml loop. Running conditions were as follows: 45°C 3 min, 45°C to 150°C at 120°C min^-1^, flow 15ml min^-1^ (He), TCD (200°C). In addition to soil gas sampling, two atmospheric air samples were collected every time and used as technical controls for quality of GC analyses. A gas mixture of 1% CO_2_, 19.8% O_2_ in N_2_ was used as a standard. Gas concentrations were expressed as % per volume.

### Soil sampling and analysis

Ten to twelve organic layer samples were collected using a soil corer (diameter 3.5 cm) from every subplot A on April 24, June 4 and August 30, 2014. Soil cores were pooled to form one composite soil sample per each subplot per sampling date, resulting in 50 soil samples per date. On average, across all blocks and treatment plots (n = 50), the humus layer was 2.55 ± 0.08 (mean ± SE) cm thick and no statistically significant differences were observed between the blocks or the treatments (F_4, 36_ = 1.9, p = 0.132). Fresh soil samples were transported to the laboratory in an icebox immediately after sampling. As soil was still frozen at the time of April sampling, samples were stored at +4°C overnight to allow slow thawing before processing. To analyze soil and microbial biomass N, one sub-sample of about 3 g soil was extracted with 50 mL of 0.5 M K_2_SO_4_. Another subsample was extracted using the same method following chloroform fumigation for 18 h [17]. The concentration of NH_4_-N was determined from fresh soil extracts according to the standard protocol (SFS 3032, Shimadzu UV-1700 spectrophotometer) and the concentration of NO_3_-N via flow analysis (FIA Perstorp). The total extractable N in both soil and fumigated extracts was oxidized to NO_3_ [18] and then analyzed as NO_3_-N (FIA, Perstorp). Microbial N was calculated by subtracting the total extractable N of the soil extracts from that of the fumigated extracts. Soil moisture was determined by drying the samples (105°C, 12 h). Soil pH was measured in 3:5 v/v soil:water suspensions (Denver Instrument Model 220).

### Seedling material and inventory of seedling condition and growth

Container seedling material for the experiment was grown by Fin Forelia Oy (Rovaniemi, Northern Finland). Seedlings of local origin were grown in PL121F (121 cells per tray, 816 cells m^-2^, cell volume 50 cm^3^) trays filled with fertilized (N, P, K and micro nutrients) and limed sphagnum peat.

One-year-old Scots pine (*Pinus sylvestris*, average height 7–12 cm) and two-year-old Norway spruce (*Picea abies*, average height 27 cm) seedlings were planted on treatment plots in September 2013 as described in Fig 1 (10 seedlings of both species in each of the 100 subplots, in total 2000 seedlings) using planting tubes to place the plug in mineral soil.

An inventory of seedling health and survival was done one month after planting on October 22, 2013 to record seedling status before winter: only 4 seedlings were visibly qualified as dead (3 pine, 1 spruce seedling, in AMB plots (2 seedlings) and COMP plots (2 seedlings)).

Seedlings in AMB, IE, COMP and NoSNOW plots were inventoried during the 2014 growing season. As soil and seedling samples in A subplots were collected in the beginning of the 2014 growing season for further biochemical analysis, only seedlings in B subplots could be inventoried. Because shelters in the NoICE plots prevented rain-on-snow but not snow compaction occurring during warm spells, the NoICE plots were considered to be similar as controls and were not used for seedling inventory. The status of seedlings was recorded on June 6 (beginning of growing season), July 11, (after cessation of the annual main shoot height growth) and September 30 (end of growing season), 2014 for the following parameters: length of the current (2014) annual shoot, health class of the current (2014) annual shoot (0: healthy, 1: <50% brown needles, 2: > 50% brown needles, and 3: dead) and seedling health class (0: healthy, 1: <50% brown needles, 2: > 50% brown needles, 3: dead).

### Statistical analysis

Data was analyzed by means of a linear mixed model, where treatment and sampling date were fixed factors and block was a random factor. Sampling date was a repeated factor with the treatment plot as a subject. Seedlings that were dead already in October 2013 were excluded from the analysis. Also seedlings with broken shoots (likely due to snow manipulation) were excluded from the dataset: a total of 10 seedlings were broken, with 8 in COMP and 2 in IE plots. For growth, seedling health and shoot winter survival, treatment-wise means per treatment plot were first computed for statistical analysis (i.e. n = 10 per treatment per sampling date). Shoots from all living seedlings, whatever their health conditions, were included when computing the mean height growth (a living seedling can have a dead shoot and a new shoot may grow the following year). Seedlings dead before winter were excluded from the dataset as well as seedlings dead in June (June growth data) and July (July growth data). Seedlings dead in July were also excluded from the September growth data as no seedling health inventory was done in September. To test whether seedlings in snow manipulation plots were statistically different from seedlings in AMB plot estimated marginal means were computed and compared under the linear mixed model. In case of the length of the current (2014) annual shoot and the proportion of dead shoots the data for the final status (Sept. 30) only was included in the analysis (i.e. date was excluded from the mixed model). Correlations were computed by means of Pearson correlation coefficients (r). All statistical analyses were carried out with SPSS 22.0 for Windows (SPSS, Inc., Chicago, IL, USA).

## Results

### Weather conditions during the experiment and snow cover data

The 2013–2014 winter was characterized by unusually warm temperatures in December, February and March (S1A Fig) with temperatures 4.1, 9.4 and 4.0°C higher than the long-term averages (1981–2010), respectively. This led to a high number of days with daily air temperatures above freezing (7, 2, 7 and 11 days in December, January, February and March, respectively, in Apukka). December 2013 was also characterized by high precipitation, mainly in the form of rain due to warm spells in early December (S1B Fig). In late winter 2014, the snow cover was thinner than the 30-year average in open areas (S1C Fig). Presence of trees and stand structure influences snow cover distribution and thermal properties due to interception of light and precipitation [14]. In the specific case of our experimental forest, we estimated that the snow cover is about half that in open area in Apukka (S1C Fig). Nevertheless, the snow depth was lower than average in winter 2013–2014 at the experimental site. Snow manipulation affected the snowpack thickness, particularly the IE treatment where a strong decrease in snow depth was measured in February (S2A Fig). The effect was lesser later in March due to the natural snow that covered the plots after the snow manipulation. The lighter snow in the NoICE plots (prevention of rain-on-snow) was the first snow to melt by the end of April (S2B Fig).

### Soil frost and temperature

**Fig 2:**
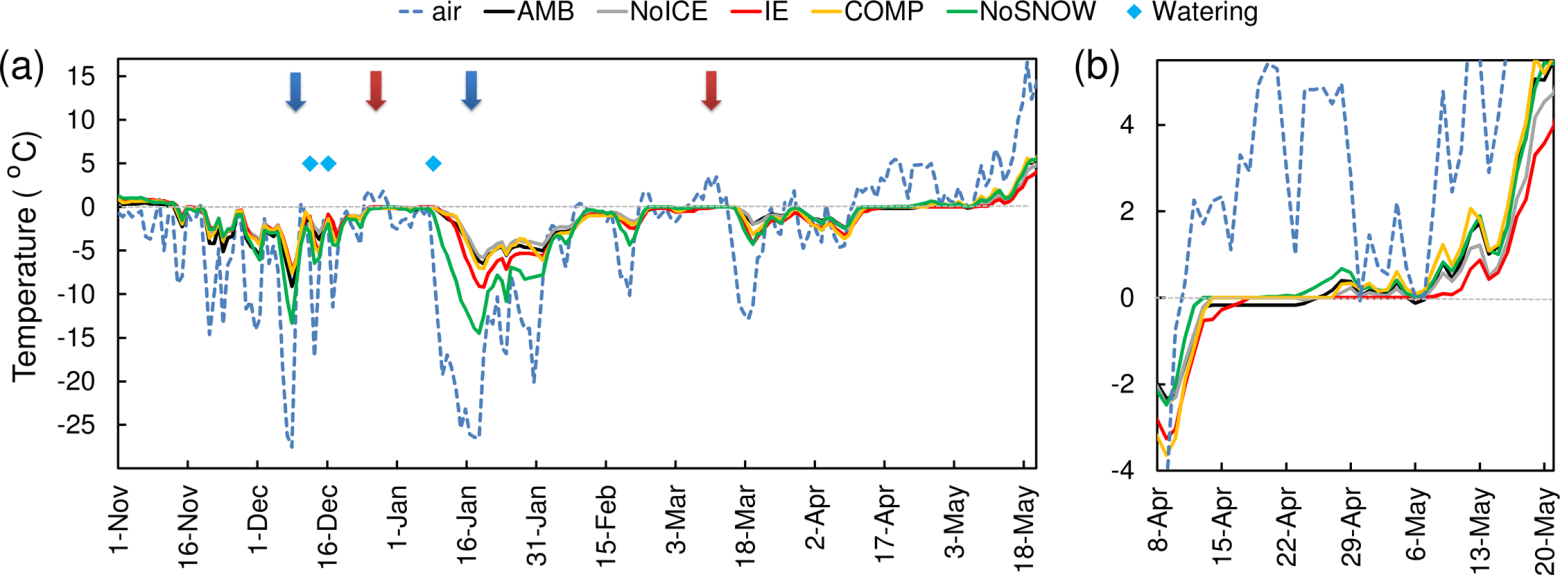
Air temperature and effect of snow manipulation on soil temperature. Mean daily air (2-m height) and soil (humus layer) temperatures during winter 2013–2014 (a) and close-up at the time of snow melt (b). Values are mean of 3 blocks. Snow watering occasions are indicated. Blue arrows = cold spells, red arrows = warms spells.

The winter 2013–2014 was characterized by two cold spells (air temperature below -20°C at the field site: December 6–9, 2013 and January 14–19, 2014) and two warm spells (air temperature above 0°C for more than 5 consecutive days, December 25–30, 2013 and March 5–14, 2014; Fig 2A). The beginning of the 2013–2014 winter was unusually warm with very little snow in early winter due to a warm spell and rain from December 25, 2013 to January 6, 2014. Due to the low snow cover in early December, the soil frost went deeper than 50 cm during the first cold spell in AMB plots (S1 Table).

The soil then completely thawed during the warm spell in early January 2014 to freeze again later in January. The warm spell in mid-March was followed by a decrease of the soil frost depth a week later (as measured on March 20, S1 Table), however not leading to complete soil thawing as the surface froze a few days after the warm spell. Subsequent freezing temperatures led to deepening of soil frost to levels similar to those before the warm spell. Finally, the soil started to thaw from the surface in the second part of April and complete soil thawing was observed in AMB plots during the third week of May (S1 Table).

During warm spells, the frost depth in NoSNOW plots decreased faster than in other plots and in late spring, the NoSNOW plots were all among the first to be completely thawed (S1 Table).

Being independent of soil physical properties, the ground surface and humus temperatures showed less heterogeneity between the blocks than soil frost. During the cold period of winter 2013–2014, snow removal, but also IE to a smaller extent, led to lower soil temperature than in AMB, NoICE and COMP plots in the humus layer (Fig 2A). However, plotting the difference in temperature between the treated and AMB plots as a function of air temperature (S3 Fig), it appeared that IE and snow compaction did not significantly decrease soil temperature under the snowpack. The natural snow that covered the plots after treatment likely provided sufficient insulation. Soil temperature in NoSNOW plots mirrored ground frost depth, and a strong correlation between the freezing air temperature and temperatures of both ground and humus were detected (r = 0.73 and 0.65, respectively; S3E Fig).

### Snow-covered area

The calculation of snow-covered area at the end of April suggested a later complete snow melt due to IE compared to ambient conditions (S2B Fig). The temperature records support a later date of snowmelt in IE compared to other plots (appearance of diurnal fluctuations in the temperature in the humus layer are linked with the date of snow melt; Fig 2B).

### Soil gas composition

**Fig 3:**
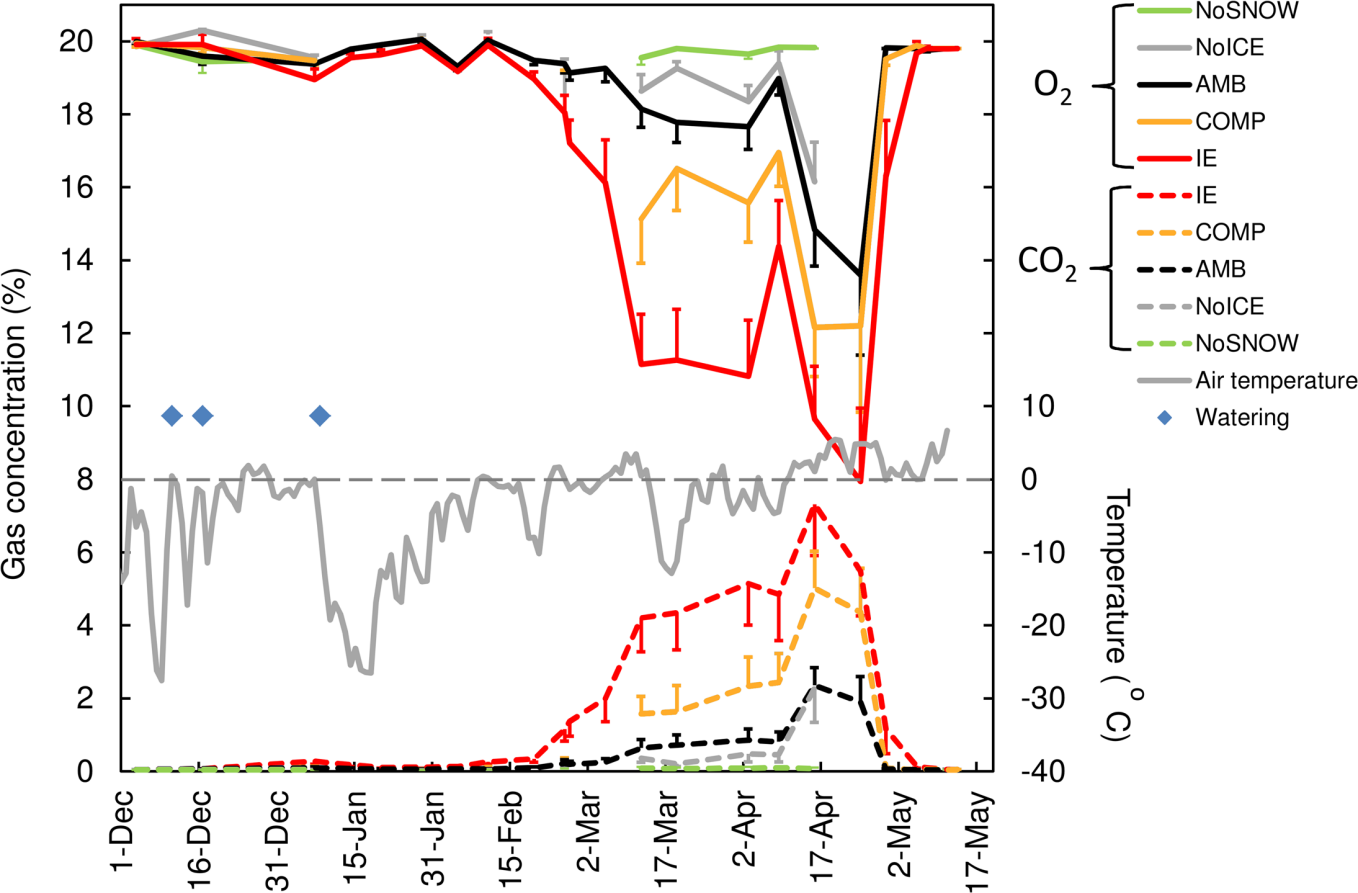
Effect of snow manipulation on CO_2_ and O_2_ concentrations 2-cm deep in humus layer. Mean daily air temperature at field site is depicted. Snow watering occasions are indicated by blue diamonds. For better clarity, one-sided error bars are depicted. Values are means ± SE (n = 10).

Analysis of gas samples collected in the humus layer (2-cm deep) throughout the winter showed large seasonal changes in CO_2_ and O_2_ concentrations and, above all, strong treatment effects (Fig 3). In AMB plots, the CO_2_ concentration started to increase from the beginning of March, up to a maximum concentration of 2.3 ± 0.5% on April 16, 2014. Similar changes were measured in the NoICE plots. Snow manipulation had a clear and statistically significant effect on soil gas composition from early March to early May (Table 1 and Fig 3).

**Table 1:** Statistical significance tests (linear mixed model for treatment, date and their interaction) for CO_2_ (log transformed) and O_2_ concentrations (%).

	log CO_2_			O_2_		
	df	F	Sig.	df	F	Sig.
Treatment	5 / 104	40.7	<0.001	5 / 162.1	19.4	<0.001
Date	22 / 520	23.3	<0.001	22 / 497.7	10.4	<0.001
Treatment*date	77 / 517	3.57	<0.001	76 /504.2	2.95	<0.001

IE led to development of hypoxia and to a strong accumulation of CO_2_ under the snowpack with concentrations of 7.3%, close to 200 times the atmospheric concentration. An intermediate situation was observed in COMP plots with concentrations between those in AMB and IE plots (Fig 3). No CO_2_ accumulation was measured in the NoSNOW plots. The arbitrary threshold of 4% (100 times the atmospheric concentration) was measured during 0, 12 and 45 consecutive days in the AMB, COMP and IE plots, respectively. Alternatively, the CO_2_ concentration was above 5% [19] during 0, 1 and 17 consecutive days in the AMB, COMP and IE plots, respectively.

The O_2_ concentration decreased in close correlation with accumulation of CO_2_ (r = -0.93). In AMB plots, the lowest O_2_ concentration was 13.7 ± 2.2%. It was lower than 16% for 9 and 41 days in the COMP and IE plots, respectively. No O_2_ decrease was observed in the NoSNOW plots. Seasonal variation in soil gas concentration under the snowpack was partly related to changes in air temperature: warm spells were followed by increases in CO_2_ concentration (decrease in O_2_ concentration) and inversely, cold periods were followed by decreases in CO_2_ concentration (increases in O_2_ concentration; Fig 3). The extreme values in April were related to the seasonal increase in air temperature in spring.

### Soil properties

Forest soil at the study site is acidic with a typical pH ranging from 3.6 and 3.9 depending on the season. Time of sampling had a significant effect on pH (S3 Table): higher pH values were measured in early June (3.85 ± 0.04) compared to April (3.77 ± 0.05) and August (3.66 ± 0.03) in AMB plots (mean ± SE, n = 10). Although treatment had no significant effect on pH (S3 Table), a tendency for lower pH was observed in IE compared to AMB plots in April and June (S4 Fig).

The snow-manipulation treatments had statistically significant effects on soil moisture and N content (S3 Table), but only the NoSNOW plots were significantly different that AMB plots. A decrease in soil moisture due to prevention of snowfall (-20% compared to AMB plots) as well as a decrease in total extractable N (the sum of NH_4_-N, NO_3_-N, and extractable organic N; S2 and S3 Tables) were detected in April but not later during the growing season. All forms of N were similarly affected by snow removal.

### Seedling winter survival

**Fig 4:**
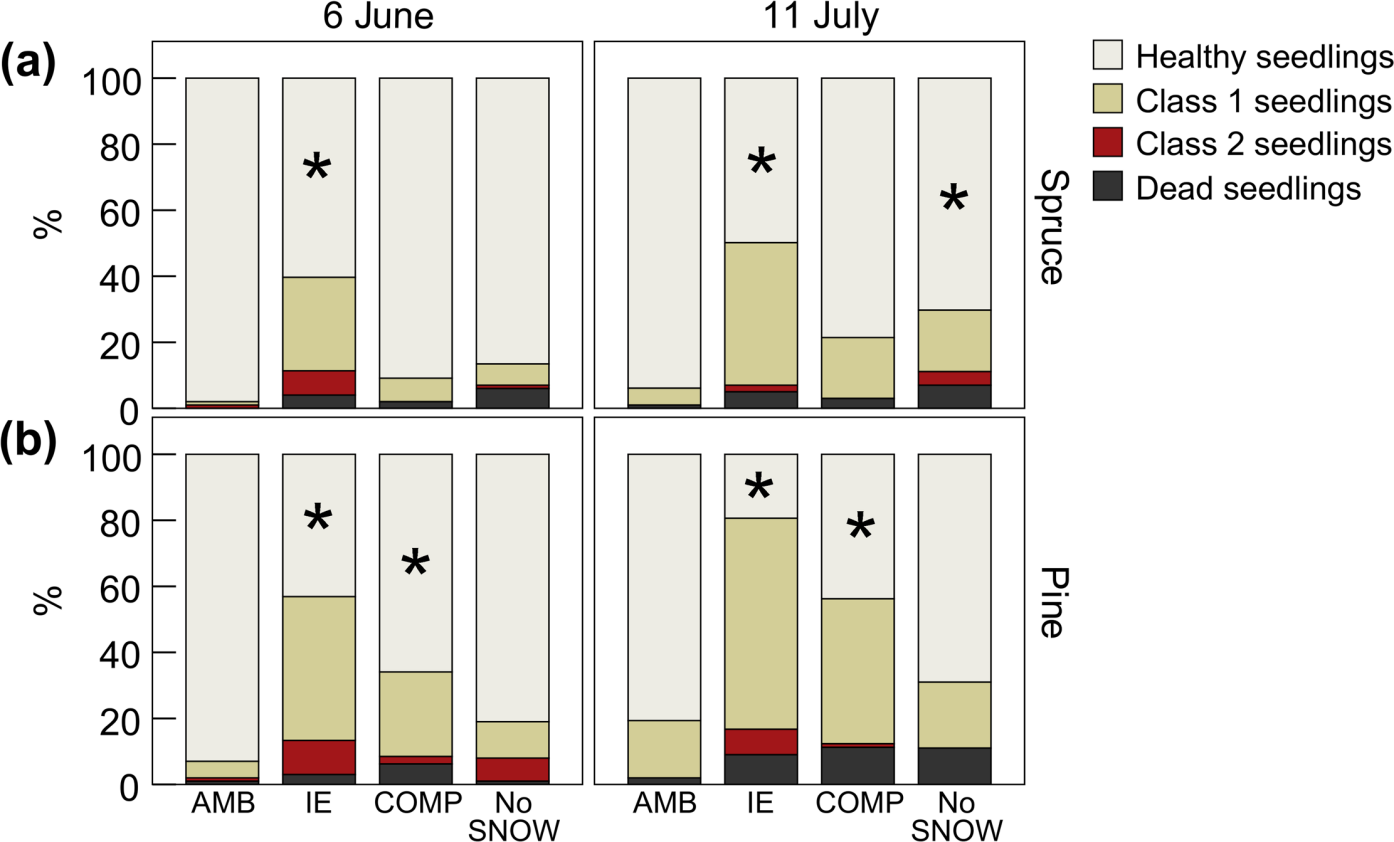
**Effect of snow manipulation on spruce (a) and pine (b) seedling survival and health the following summer.** Seedlings were inventoried on June 6 and July 11, 2014. Class 1: <50% brown needles, class 2: > 50% brown needles. An asterisk indicates a statistically significant difference in proportion of healthy seedlings with AMB plots at p < 0.05. Values are means (n = 10).

In general, seedling winter survival was high in ambient conditions (AMB plots) with 94% of spruce seedlings and 80% of pine seedlings being healthy in mid-July. One winter of snow manipulation significantly affected the survival of seedlings (Fig 4 and Table 2). IE was the most damaging treatment with only 50% of spruce and 19% of pine seedlings still healthy in mid-July. Compared to the situation in AMB plots, those values represent 47% less spruce and 76% less pine seedlings healthy. Likewise, COMP was less damaging to spruce than pine (79% of spruce and 49% of pine seedlings were healthy in July) but the NoSNOW treatment affected both species similarly (70% of spruce and 69% of pine seedlings were healthy in July).

**Table 2:** Statistical significance tests (linear mixed model for treatment, date and their interaction) for proportion of healthy spruce and pine seedlings in June and July.

	Spruce			Pine		
	df	F	Sig.	df	F	Sig.
Treatment	3 / 60.25	19.03	<0.001	3 / 57.69	38.19	<0.001
Date	1 / 60.25	7.47	0.008	1 / 57.69	19.8	<0.001
Treatment*date	3 / 60.25	0.41	0.745	3 / 57.69	0.63	0.60

The proportion of dead seedlings did not change significantly until mid-July for spruce seedlings but increased from June to mid-July for pine seedlings (16 spruce and 34 pine seedlings were dead in mid-July). This was particularly true in NoSNOW plots where more than 90% of the dead pine seedlings in July died between early June and mid-July (Fig 4). None of the heavily damaged spruce seedlings (class 2) recorded in June, including those in IE plots, died within the next month and most of them recovered to a better health class (7 out of 9 seedlings, among which 7 were in IE plots). The situation was different for pine, as among the 19 class 2 seedlings inventoried in June (10 in IE plots); 50% died during the next month and only 4 improved in health.

### Current-year shoot survival and growth

**Fig 5:**
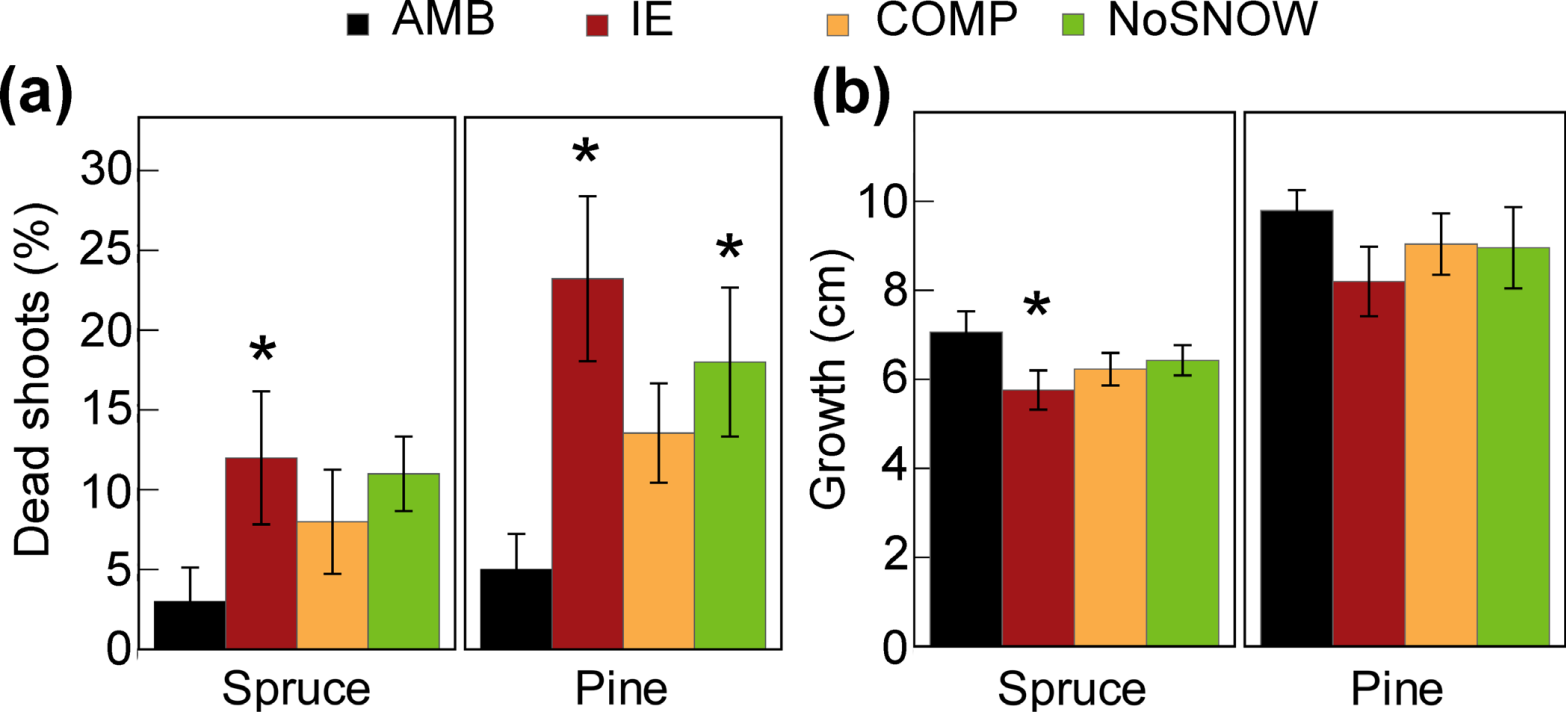
**Effect of snow manipulation on main shoot winter survival (a) and shoot growth (b) at the end of the growing season (September 30, 2014).** An asterisk indicates statistically significant difference with AMB plots at p < 0.05. Values are means ± SE (n = 10).

All treatments tended to increase the number of dead shoots compared to AMB conditions but in case of spruce only IE, and in case of pine IE and No SNOW were significantly different from AMB (Fig 5A, S5 Table). In both species, almost all the shoots dead at the end of the growing season were already dead in early June (S4 Table). Seedlings in IE, COMP and NoSNOW treatments tended to grow less than seedlings in the ambient conditions (Fig 5, S5 Table). At the end of the growing season the height growth of spruce seedlings was significantly (p = 0.004) lower that in the ambient, and in the COMP marginally so (p = 0.054). In case of pine the height growth in the IE was only marginally (p = 0.08) lower than in ambient. Measurement of terminal shoot length in July and September showed that all seedlings had completed their annual height growth by mid-July and that treatments did not cause any delay in growth (S4 Table).

## Discussion

In line with our hypothesis, altered snowpack had strong consequences on the overwintering conditions of plants and conifer seedling survival. As expected, IE but as well snow compaction limited gas exchange at the soil-atmosphere interface, inducing development of hypoxia and accumulation of CO_2_ in the subnivean environment. Accumulation of CO_2_ was detected also in ambient plots (AMB), and in NoICE plots to a lesser extent, but the major consequence of IE was a higher maximum CO_2_ concentration (7.3%, more than three times higher than in AMB plots) and a longer duration of high CO_2_ concentration. The 5% threshold for induction of growth and metabolic symptoms in the case of flooded roots [19] was exceeded for 17 consecutive days in IE plots but not recorded at all in AMB plots in the 2013–2014 winter. In contrast to our hypothesis, snow compaction and IE had no clear effect on soil temperature but showed only a tendency of lower temperatures compared to AMB plots during the coldest periods. This result is likely due to the natural snow that covered the plots after snow manipulation in January. By contrast, snow removal exerted a considerable influence on soil temperatures and removal of the insulating snow cover led to deeper soil frost as previously described [20–23]. However, deeper soil frost did not delay soil warming during warm spells or soil thawing in spring, likely due to fast soil-atmosphere heat exchange and low albedo due to lack of snow cover. Consistent results have been previously reported in a complete snow removal experiment in a boreal forest site [20]. Instead, partial snow removal that maintained albedo similar to reference plots led to extension of soil frost duration [24, 25]. Our results also indicated a delay in snow ablation due to IE which is in agreement with Rixen et al. [26] who reported a positive correlation between date of snow melt and snow density. We also detected a tendency of lower pH in IE conditions compared to AMB plots in April. A decrease in pH in relation to CO_2_ accumulation has been previously measured in natural CO_2_ vents [27] or after CO_2_ injection in soil [28]. However, the reported concentrations were much higher than the values reported in our study and whether the small drop in pH we measured is a consequence of CO_2_ accumulation during winter remains to be proven.

Microbial, faunal and plant root respiration that decrease O_2_ and increase CO_2_ concentrations in the soil environment continue over the winter under the snowpack where temperatures are maintained close to zero throughout the winter [29]. Particularly, soil microbial respiration may constitute a major wintertime source of CO_2_, because soil microorganisms in cold environments are adapted to efficiently function at close-to-zero or even sub-zero temperatures [30, 31]. Higher CO_2_ concentrations in the organic layer in the plots receiving IE or snow compaction treatments could either be related to a decrease in CO_2_ soil efflux due to the trapping of gases under the ice layer and in the frozen soil, or to higher microbial production of CO_2_. Although we did not directly analyzed the effects of the changing subnivean environment on soil microbial activity, increasing CO_2_ production under IE and compacted snow are most likely not due the minimal or even cooling effect on soil temperature. Soil microbial activity is also governed by carbon availability to soil microorganisms and we cannot exclude that the possible higher fine root mortality due to snow manipulation (see below) has an impact on soil microbial activity. However, we suggest that the kinetics of soil gas efflux through the snowpack, rather than CO_2_ production, were affected in IE conditions due to the hermetic ground-ice layers and the efficiency of ice in trapping gases inside the soil environment. This hypothesis is supported by the large flux of CO_2_ measured at the time of snowmelt [32] and finding that microbial activity during the following growing season was lower in the IE treated-plots (S. Stark, unpublished). Accumulation of CO_2_ in soil due to limited soil-atmosphere gas exchange under snow cover has been previously measured even at the depth of 35 cm in montane soil [12] and in high elevation forest soil [33]. The maximum CO_2_ concentrations reported in our study in ambient conditions are higher than the values reported in these previous studies. This likely resulted from the wet weather conditions during winter 2013–2014 and the naturally occurring IE. Previously, Yanai et al. (2011) reported development of hypoxia under the snowpack but they did not monitor CO_2_ concentrations [34].

In line with our hypothesis, changes in the snow-cover significantly affected winter survival and growth of conifer seedlings. In general, the survival rate of conifer seedlings was high with 94% of spruce and 80% of pine seedlings being healthy in mid-July in ambient conditions. IE induced the most damaging effect with only 50% of spruce and 19% of pine seedlings still healthy in mid-July. Only IE showed significant effect on height growth. Contrary to other studies [24, 35], growth was not significantly affected by snow removal. Increased shoot mortality in response to IE was previously found in *Vaccinium* species, but not in a dominant evergreen dwarf shrub in boreal systems, *Empetrum nigrum*, suggesting high species-specificity in plant responses to IE [36]. Soil gases were not however monitored in that study. The same authors exposed the above-ground part of the same species to hypoxia or high CO_2_ concentrations within the snowpack for 14 days to simulate ice layer on top of the snowpack and species-specific symptoms were observed in response to high CO_2_ only [37].

Because snow manipulations changed the environmental conditions for conifer seedlings in multiple ways (e.g. low freezing soil temperature, early soil thaw due to complete snow removal, hypoxia, high CO_2_ accumulation and delayed snow melt due to IE), it is difficult to determine which of these factors had the most important influence on seedling survival and growth. Both direct and indirect mechanisms are probably involved in the different treatment effects. Nitrogen availability was unaffected by IE or snow compaction, so it cannot explain the symptoms observed under those snow conditions. Winter–induced symptoms in boreal zone are typically related to the effect of temperature such as frost damage, winter desiccation or photoinhibition [38]. Without any protective snow cover, seedlings are more exposed to freezing temperature, freeze-thaw cycles and direct sun light which typically lead to stronger photoinhibition than in seedlings under the snow cover [39]. As we did not measure photochemical efficiency in this study, we cannot evaluate the role of photoinhibition in the damages observed due to snow manipulation, and particularly snow removal. However, we suggest that in absence of snow, lower soil temperature is a determinant factor leading to damage. Low soil temperature in early winter did not affect the seedlings in AMB plots (-10°C minimum soil temperature in early December) suggesting that early-winter low freezing temperatures do not induce significant abiotic stress for the dormant seedlings but that soil frost later in winter is more critical, in relation to freeze-thaw cycles and root damages in snow free soil [40]. Furthermore, seedlings outplanted in late autumn do not grow roots before winter (Luoranen, unpublished), which might increase the risk of damage during snowless winter or dry spring, as it is the case in absence of snow. A snow removal experiment for two consecutive winters showed that Norway spruce seedlings heavily suffered from winter desiccation and had lower growth during the following summer [35]. Unfortunately root biomass was not investigated. Partial snow removal also increased root exposure and decreased post-winter survival of saplings from different species, the effects being species-specific [41]. More specifically, complete or partial snow removal has been shown to increase fine root mortality in trees by direct cellular damage and indirect damage to the roots by mechanical processes related to freeze-thaw cycles and consequent frost heaving [24, 40, 42–44]. Reduced terminal bud growth usually reflects root growth and root damage [24], which could explain the growth reduction detected in the present study. The effects are however expected to depend on tree age (seedlings versus mature trees). For example, deep soil freezing after snow removal was not detrimental for fine roots of mature Norway spruce [45], but for spruce seedlings soil freezing has been shown to affect shoot and root growth in the following growing season [46, 47]. In Scots pine seedlings, freezing soil has been found to strongly increase root mortality [43]. In both species, damage is likely not caused by direct exposure to low temperatures but rather the consequence of dehydration. In that sense, the duration but as well the intensity of frost appear as critical parameters and so the rooting depths of seedlings versus adult trees.

IE induced more detrimental effects on conifer seedlings than snow removal, and its effect on seedling and shoot health appeared earlier during the growing season. Ice encased seedlings experienced slightly lower soil temperatures than those in ambient conditions during cold spells but, more importantly, they experienced hypoxic conditions with high CO_2_ concentrations under the ice layers. Snow compaction led to concentrations of soil gases and to seedling and shoot health damage at intermediate levels between IE and ambient conditions. Plant response to hypoxia is well known in connection with waterlogging and flooding [48, 49], but, compared to waterlogged conditions, IE is formed at below or near freezing soil temperatures when trees have a low metabolic status. Low oxygen demand in dormant trees may partly mitigate the harmful effects of overwintering under ice layers [48] but, on the other hand, dormant trees have no or limited photosynthesis to allow for energy supply to the hypoxic roots. The effect of high soil CO_2_ concentration on plants has been studied, e.g. in connection with the development of carbon capture and storage (CCS) technology. For example, CO_2_ injection into soil during the growing season led to leaf discoloration in several crops species within a week [50]. An increased of CO_2_ concentration in soil from 0.035% to 0.7% has been shown to decrease Douglas fir root respiration by 50% [51], but generally very little information is available on the impact of soil CO_2_ on the roots of woody plants. The effects of high soil CO_2_ on plants depend mainly on root characteristics (root length, porosity and resistance to CO_2_ movement across exodermis) [19]. At the cellular level, an increase in internal CO_2_ partial pressure has been shown to lead to cytoplasm acidification, running the risk of adverse effects on metabolism or cell structure [52], and consequently root functioning. However, the water and nutrient uptake that is coupled to active CO2 uptake in e.g. flooded roots are nonexistent or low in winter months and make this winter situation typical. Hypoxia and CO_2_ accumulation are tightly linked and whether hypoxia or high CO_2_ concentration is the most damaging agent behind the observed seedling damage remains to be proven [19]. Castonguay et al. [53] exposed perennial grasses to different O_2_ and CO_2_ concentrations during winter. They observed that low O_2_ concentration reduced regrowth more than high CO_2_ alone, but a combination of high CO_2_ and low O_2_ concentrations seemed to be more damaging than low O_2_ alone. The exposure of above ground part of sub-arctic dwarf shrubs to either high CO_2_ concentration or hypoxic conditions in the snowpack for 14 days revealed that CO_2_ alone caused damages, whereas dwarf shrubs were able to tolerate hypoxia (low O_2_ alone) [37]. In this later study, responses were however species-specific. Although different underlying mechanisms are hypothesized to explain the difference between IE and snow removal effects, one major difference between those treatments is the conditions seedlings experienced at the very beginning of the growing season. Date of snowmelt is indeed essential for plant growth as it regulates soil moisture [54] and nutrient availability. In spring, both air and soil temperatures trigger the beginning of the active growing period [55, 56]. In our study, snow removal led to low soil moisture, early soil thaw and consequently to higher risk of drought and spring frost damage (last frost observed on May 15, 2014). In contrast, IE led to later soil thaw and snow melt, which represent a high risk of winter desiccation due to limited liquid water availability in frozen soil and aboveground conditions allowing photosynthesis resumption in seedlings emerging from the thin snow cover. Excessive delay in soil thawing compared to the increase in air temperature in spring can impair growth of above- and below-ground organs of trees and potentially lead to death [20, 45, 57, 58]. The severity of stress also depends on the length of the delay in thawing [57]. So, both snow removal and IE led to highly stressful conditions at the time of spring regrowth which could affect growth but potentially survival as well. Our results also suggest that all three snow manipulation levels have more significant effects on seedling survival and health than on growth. Whether growth reduction is the direct consequence of health weakening remains to be studied. For example, different environmental factors, date of snowmelt and elevation, appear to be determinant for survival and growth, respectively, of seedlings planted above the Alpine treeline [59].

Among IE, snow compaction or absence of snow, IE appeared to be the most harmful winter conditions to both conifer species. The frequency of warm spells and rain-on-snow events causing snow compaction and ice layers under or within the snowpack are projected to increase in the future [3]. The total absence of snow cover is outside the range of natural variation in Northern Finland (66°N) but is part of the anticipated climate change variation in Southern Finland [4]. Our results revealed for the first time that by modifying the snow conditions in boreal forests, changing winter climate might have adverse effects on growth and winter survival of conifer seedlings. These effects could influence forest regeneration with important implications for boreal forest ecology and the associated economy. This is particularly true for afforestation as planting costs represented 58 million euros for 84000 ha, 2/3 of the regenerated forest area in Finland in 2012 [60]. Although further studies are needed to determine which of the winter stresses caused the most damaging effects, our results suggest that denser snowpack or lack of snow cover due to winter warming could have a major impact on forest ecosystems. Understanding the physiological mechanisms behind the observed seedling decline and death are needed in order to find tools for mitigating the effect of IE on boreal forest ecosystem functioning. So far, models forecasting growth and productivity of boreal forest under climate change scenarios only consider changing conditions during the growing season. Our results indicate that the possible consequences of winter climate change should be taken into consideration in these models. Winter climate change might have a negative effect on forest growth and productivity that could partially counteract the positive growth effects due to increasing growing season temperatures.

## Supporting Information

S1 Fig 1981–2010 average and winter 2013–2014 air temperature, precipitation and snow depth. (PDF)

S2 Fig Effect of snow manipulation on snow depth at field site and snow-covered area. (PDF)

S3 Fig Effect of snow manipulation on ground and humus temperatures. (PDF)

S4 Fig Effect of snow manipulation on pH of humus layer. (PDF)

S1 Table Effect of snow manipulation on soil temperature, depth of soil frost and snow ablation. (PDF)

S2 Table Effect of snow manipulation on soil moisture and N content. (PDF)

S3 Table Statistical significance tests for soil properties. (PDF)

S4 Table Effect of snow manipulation on main shoot winter survival and growth. (PDF)

S5 Table Statistical significance tests for dead main shoot and seedling growth. (PDF)
