# Supplementary material for: The Snow Must Go On: Ground Ice Encasement, Snow Compaction and Absence of Snow Differently Cause Soil Hypoxia, CO2 Accumulation and Tree Seedling Damage in Boreal Forest
Source: PLoS One. 2016 Jun 2;11(6):e0156620. doi: 10.1371/journal.pone.0156620 (PMC4890806; doi:10.1371/journal.pone.0156620)

S1 Fig. 1981-2010 average and winter 2013-2014 air temperature (a), precipitation (b) and snow depth (c) in Apukka (WMO field station 02813), 15km N of Rovaniemi, and at the experimental forest site. The snow curve 1981-2010 at the forest site is an estimation derived from Apukka data (open area) and snow depth measurements made at the experimental site in winters 2015 and 2016 to estimate the local canopy and stand structure effects on snow cover.

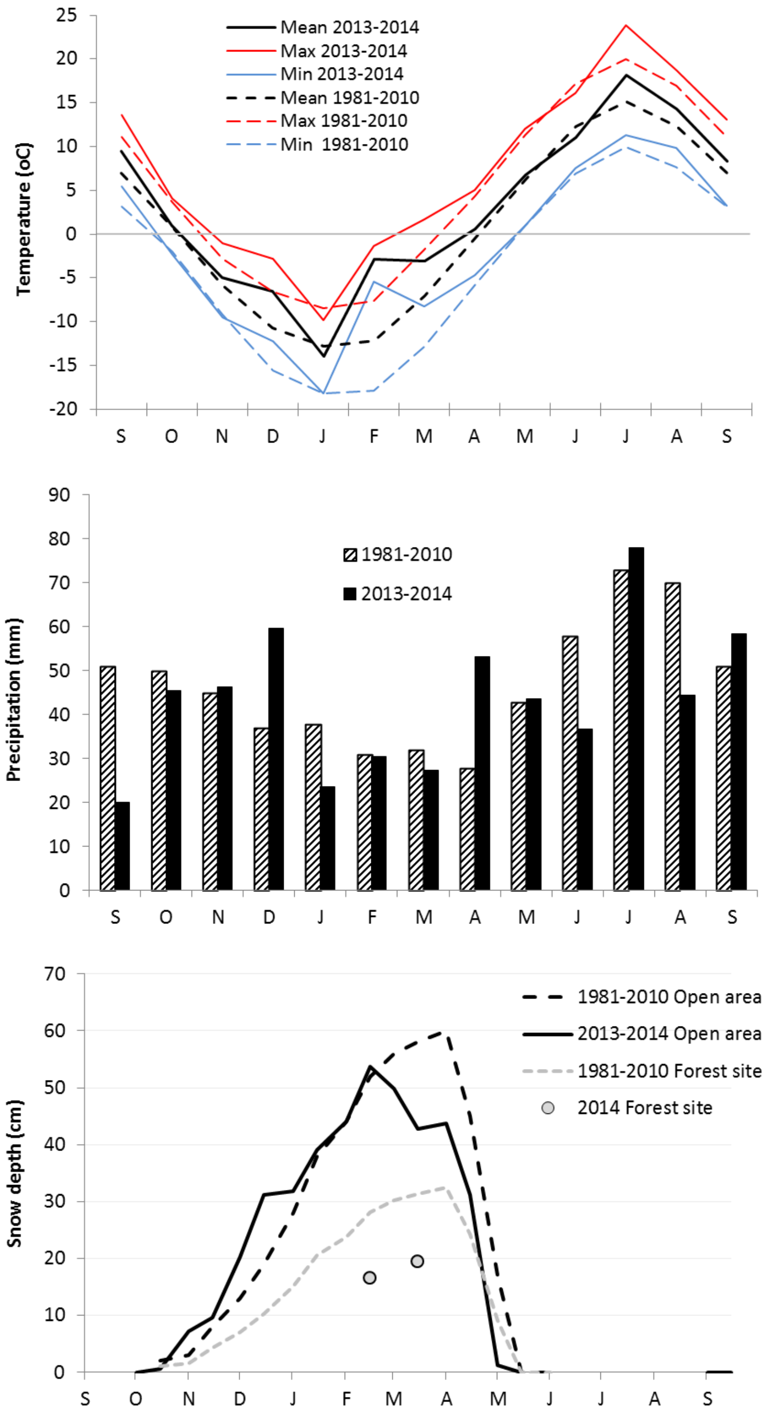

Supplement: S1 Fig — (PDF) [file pone.0156620.s001.pdf]
