# Supplementary material for: The Snow Must Go On: Ground Ice Encasement, Snow Compaction and Absence of Snow Differently Cause Soil Hypoxia, CO2 Accumulation and Tree Seedling Damage in Boreal Forest
Source: PLoS One. 2016 Jun 2;11(6):e0156620. doi: 10.1371/journal.pone.0156620 (PMC4890806; doi:10.1371/journal.pone.0156620)

S2 Fig. Effect of snow manipulation on snow depth at field site (n = 3) (a) and snow-covered area on April 24, 2014 (n = 10) (b). Values are means ± SE.

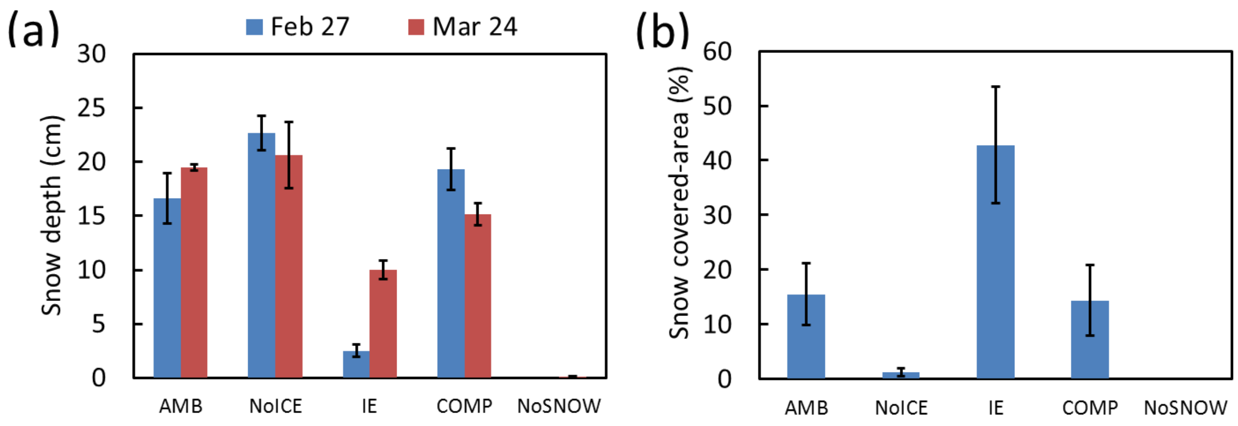

Supplement: S2 Fig — (PDF) [file pone.0156620.s002.pdf]
