# Supplementary material for: The Snow Must Go On: Ground Ice Encasement, Snow Compaction and Absence of Snow Differently Cause Soil Hypoxia, CO2 Accumulation and Tree Seedling Damage in Boreal Forest
Source: PLoS One. 2016 Jun 2;11(6):e0156620. doi: 10.1371/journal.pone.0156620 (PMC4890806; doi:10.1371/journal.pone.0156620)

S3 Fig. Effect of snow manipulation on ground and humus temperatures. Relation between the air and soil temperatures in AMB plots (a) and difference between NoICE and AMB (b), IE and AMB (c), COMP and AMB (e) and NoSNOW and AMB (d) plots as a function of air temperature. Negative values show cooling effect.

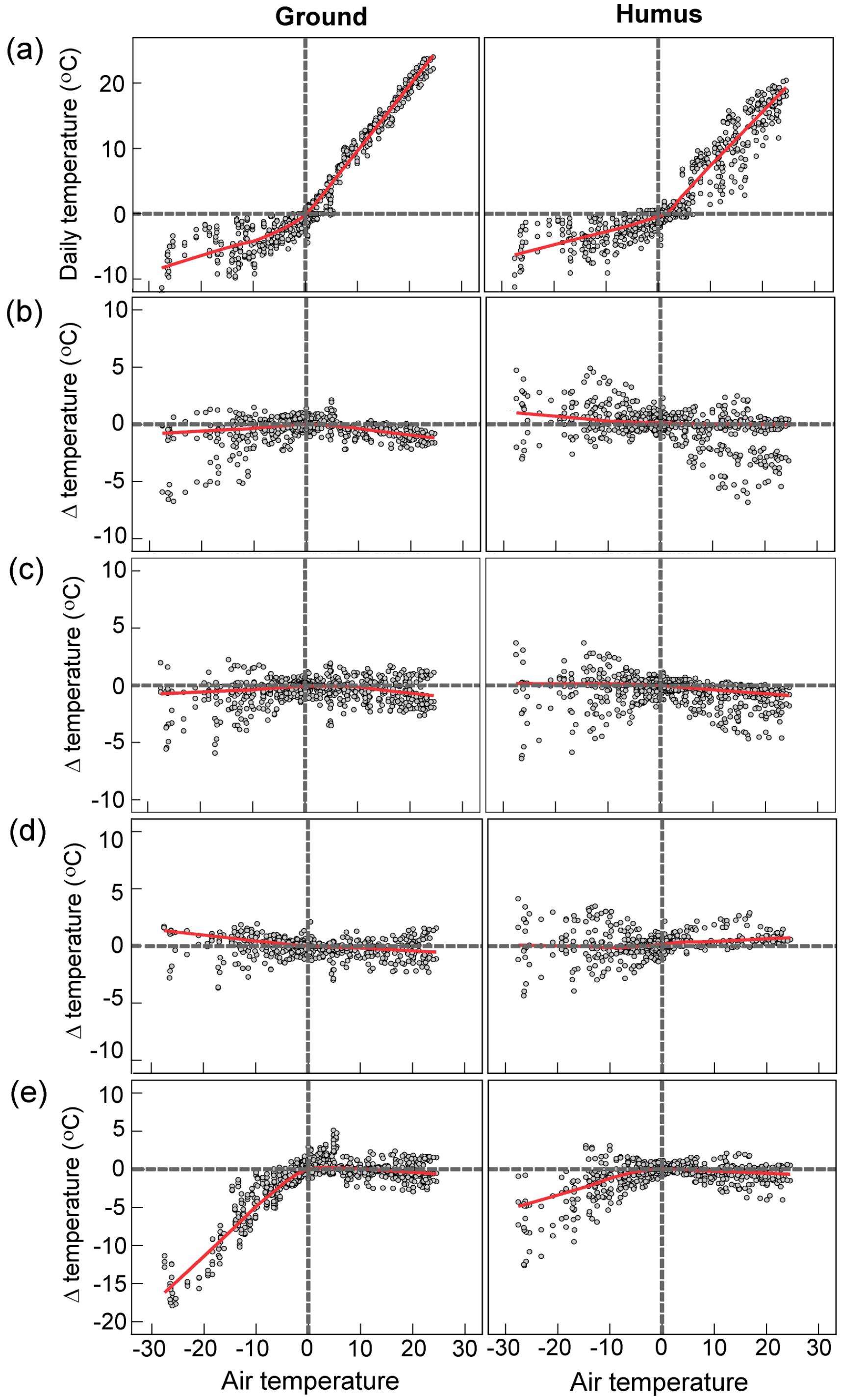

Supplement: S3 Fig — (PDF) [file pone.0156620.s003.pdf]
