# Supplementary figures and images for: The Snow Must Go On: Ground Ice Encasement, Snow Compaction and Absence of Snow Differently Cause Soil Hypoxia, CO2 Accumulation and Tree Seedling Damage in Boreal Forest
Source: PLoS One. 2016 Jun 2;11(6):e0156620. doi: 10.1371/journal.pone.0156620 (PMC4890806; doi:10.1371/journal.pone.0156620)

**S4 Fig. Effect of snow manipulation on pH of the humus layer.** Values are means  $\pm$  SE (n=10).

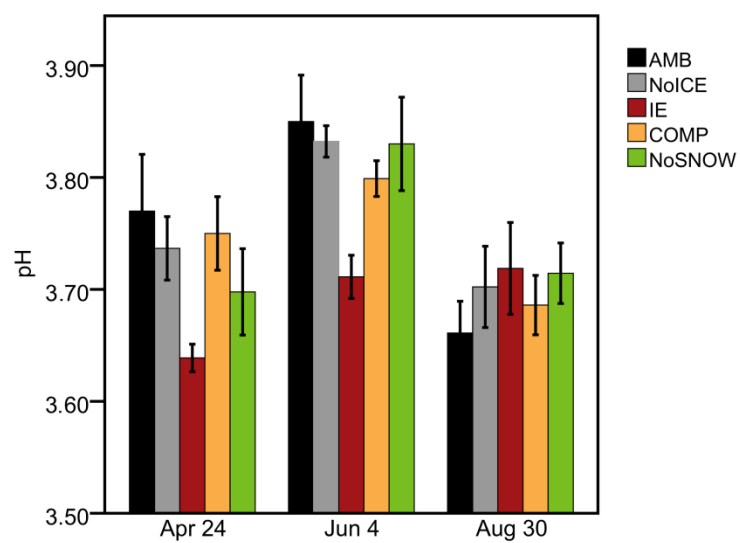

Supplement: S4 Fig — (PDF) [file pone.0156620.s004.pdf]
