# Supplementary material for: The Snow Must Go On: Ground Ice Encasement, Snow Compaction and Absence of Snow Differently Cause Soil Hypoxia, CO2 Accumulation and Tree Seedling Damage in Boreal Forest
Source: PLoS One. 2016 Jun 2;11(6):e0156620. doi: 10.1371/journal.pone.0156620 (PMC4890806; doi:10.1371/journal.pone.0156620)
Supplement: S1 Table — (PDF) [file pone.0156620.s005.pdf]

S1 Table: **Effect of snow manipulation on depth of soil frost and timing of snow ablation.**

Block	Treatment	Cold spell 9.12.2013 surface temp.	Cold spell 9.12.2013 humus temp.	Cold spell Frost line 17.12.13 *	Warm spell 29.12.2013 surface temp.	Warm spell 29.12.2013 humus temp.	Warm spell Frost line 9.1.14 *	Cold spell 19.1.2014 surface temp.	Cold spell 19.1.2014 humus temp.	Cold spell Frost line 11.2.14 *	Warm spell 12.3.2014 surface temp.	Warm spell 12.3.2014 humus temp.	Warm spell Surface thawing 13.3.14*	Warm spell Frost line 20.3.14 *	Complete soil thawing**	Snow ablation Mean daily temp.>0 surface temp.	Snow ablation Mean daily temp.>0 humus	Snow ablation Surface thawing **	Snow covered-area 24.4.14
		°C	*°C*	*cm*	*°C*	*°C*	*cm*	*°C*	*°C*	*cm*	*°C*	*°C*	*cm*	*cm*	*date*	*date*	*date*	*date*	*%*
**4**	AMB	-12.2	-11.2	54	0.0	0.0	1	-9.3	-8.9	88	0.0	0.0	5	77	12.5.	19.4.	23.4.	25.4.	9.2
	NoICE	-18.1	-6.5	42	0.0	0.0	0	-15.3	-6.0	67	0.0	0.0	19	14	28.5.	18.4.	15.5.	8.5.	2.5
	IE	-10.3	-7.5	46	0.0	0.0	0	-9.8	-8.7	83	0.0	0.0	0	77	10.6.	26.4.	10.5.	22.5.	94.1
	NoSNOW	-25.1	-15.5	65	1.1	0.0	0	-24.3	-16.5	103	1.4	0.0	27	45	12.5.	10.4.	23.4.	25.4.	3
	COMP.	-10.5	-7.1	35	0.0	-0.5	0	-8.0	-6.5	74	0.0	na	0	70	3.6.	24.4.	na	15.5.	20.5
**5**	AMB	-12.1	-6.8	42	0.0	-0.5	0	-6.3	-3.3	60	0.0	0.0	0	45	17.5.	24.4.	12.5.	5.5.	52.3
	NoICE	-12.3	-8.8	51	0.0	0.0	0	-7.7	-6.3	62	0.0	0.0	0	50	5.5.	12.4.	29.4.	5.5.	na
	IE	-12.7	-7.3	49	0.0	0.0	4	-11.9	-9.6	81	0.0	0.0	0	73	5.5.	20.4.	10.5.	25.4.	20.7
	NoSNOW	-23.5	-14.1	73	1.0	-0.1	46	-23.4	-15.8	111	2.1	0.0	14	37	5.5.	11.4.	25.4.	16.4.	0
	COMP.	-10.5	-8.0	43	0.0	0.0	0	-9.1	-7.6	74	0.0	0.0	0	50	28.5.	23.4.	27.5.	5.5.	22.5
**10**	AMB	-11.3	-9.4	34	0.0	0.0	0	-7.2	-4.8	50	0.0	0.0	0	15	22.5.	23.4.	27.5.	5.5.	32.6
	NoICE	-11.4	-7.1	33	0.0	0.0	2	-7.7	-9.0	49	0.0	0.0	2	40	17.5.	20.4.	27.4.	25.4.	1.2
	IE	-11.5	-6.7	35	0.0	0.0	0	-10.9	-11.1	46	0.0	0.0	0	38	12.5.	25.4.	7.5.	5.5.	65.7
	NoSNOW	-23.5	-10.3	38	1.2	0.0	8	-22.6	-7.0	46	1.9	0.0	11	9	5.5.	10.4.	23.4.	25.4.	0
	COMP.	-9.7	-7.7	42	na	0.0	0	na	na	53	na	na	27	8	28.5.	na	na	5.5.	3.5

Frost tubes and temperature loggers were installed in three randomly selected blocks in autumn 2013.

* : data from frost tubes

** : Closest date of frost tube reading
