# Supplementary material for: The Snow Must Go On: Ground Ice Encasement, Snow Compaction and Absence of Snow Differently Cause Soil Hypoxia, CO2 Accumulation and Tree Seedling Damage in Boreal Forest
Source: PLoS One. 2016 Jun 2;11(6):e0156620. doi: 10.1371/journal.pone.0156620 (PMC4890806; doi:10.1371/journal.pone.0156620)
Supplement: S2 Table — (PDF) [file pone.0156620.s006.pdf]

S2 Table: Effect of snow manipulation on soil moisture and N content in the humus layer in 2014.

Date	Treatment	Moisture (%) (%)	N mic. (mg/kg DW)	N org. (mg/kg DW)	N total (mg/kg DW)	NH4-N (mg/kg DW)	NO3-N (mg/kg DW)	OM (% DW)
24 Apr	AMB	75.0 ± 1.8	208.7 ± 24.3	123.7 ± 7.8	141.4 ± 11.0	8.28 ± 2.47	1.80 ± 0.31	62.5 ± 5.5
	NoICE	72.8 ± 2.5	210.7 ± 19.3	152.5 ± 25.7	163.3 ± 24.2	7.84 ± 1.70	1.64 ± 0.23	60.6 ± 5.0
	IE	75.3 ± 2.2	242.9 ± 27.7	145.1 ± 20.7	153.6 ± 22.7	6.97 ± 2.43	1.61 ± 0.28	65.6 ± 5.4
	COMP	75.2 ± 1.3	194.5 ± 15.3	141.8 ± 14.5	148.8 ± 14.0	5.57 ± 1.86	1.43 ± 0.15	63.3 ± 4.2
	NoSNOW	59.6 ± 2.0	98.4 ± 13.6	59.9 ± 8.8	62.7 ± 8.5	2.06 ± 0.51	0.66 ± 0.13	29.5 ± 2.6
2 Jun	AMB	44.8 ± 2.3	142.8 ± 18.6	54.0 ± 4.6	60.4 ± 5.0	5.60 ± 0.58	0.83 ± 0.05	35.0 ± 2.9
	NoICE	44.4 ± 1.9	120.9 ± 9.3	54.8 ± 6.5	66.3 ± 8.5	4.52 ± 0.36	0.83 ± 0.05	35.6 ± 3.3
	IE	48.6 ± 2.3	148.3 ± 14.8	66.8 ± 7.2	73.1 ± 7.7	5.43 ± 0.60	0.93 ± 0.07	42.8 ± 4.4
	COMP	46.1 ± 1.9	153.2 ± 14.0	57.1 ± 4.0	63.2 ± 4.5	5.27 ± 0.50	0.80 ± 0.04	37.3 ± 3.2
	NoSNOW	44.6 ± 2.1	140.4 ± 12.6	61.4 ± 5.3	67.7 ± 5.6	5.48 ± 0.41	0.81 ± 0.06	37.0 ± 3.6
26 Aug	AMB	43.0 ± 2.1	162.6 ± 20.3	51.5 ± 8.1	56.5 ± 8.5	3.53 ± 0.41	1.38 ± 0.14	47.7 ± 4.8
	NoICE	41.6 ± 2.8	140.8 ± 11.9	42.3 ± 4.7	52.1 ± 5.5	3.83 ± 0.52	1.13 ± 0.05	40.8 ± 3.5
	IE	44.3 ± 2.4	178.0 ± 14.9	49.9 ± 4.2	54.8 ± 4.4	3.59 ± 0.26	1.28 ± 0.12	48.5 ± 4.5
	COMP	39.4 ± 2.1	152.2 ± 7.1	52.0 ± 3.2	56.5 ± 3.4	3.29 ± 0.33	1.18 ± 0.06	41.7 ± 2.5
	NoSNOW	44.1 ± 2.7	165.1 ± 13.1	54.2 ± 4.6	59.7 ± 4.7	4.24 ± 0.39	1.27 ± 0.08	44.5 ± 3.2

Values are means ± SE (n=10).
