# Supplementary material for: The Snow Must Go On: Ground Ice Encasement, Snow Compaction and Absence of Snow Differently Cause Soil Hypoxia, CO2 Accumulation and Tree Seedling Damage in Boreal Forest
Source: PLoS One. 2016 Jun 2;11(6):e0156620. doi: 10.1371/journal.pone.0156620 (PMC4890806; doi:10.1371/journal.pone.0156620)
Supplement: S3 Table — (PDF) [file pone.0156620.s007.pdf]

S3 Table: Statistical significance tests for soil moisture (%), total N concentration (log transformed mg kg DW^{-1}) and pH.

	Moisture			Log N totdw			pH		
Source	df	F	Sig.	df	F	Sig.	df	F	Sig.
Treatment	4 / 42.1	3.71	0.011	4 / 39.8	3.53	0.015	4 / 33.6	0.063	0.992
Date	2 / 91.3	322.4	<0.001	2 / 85.6	110.92	<0.001	2 / 44.8	28.35	<0.001
Treatment*date	8 / 91.3	4.73	<0.001	8 / 85.6	7.74	<0.001	8 / 44.7	1.92	0.081

Linear mixed model for treatment, date and their interaction.
