# Supplementary material for: The Snow Must Go On: Ground Ice Encasement, Snow Compaction and Absence of Snow Differently Cause Soil Hypoxia, CO2 Accumulation and Tree Seedling Damage in Boreal Forest
Source: PLoS One. 2016 Jun 2;11(6):e0156620. doi: 10.1371/journal.pone.0156620 (PMC4890806; doi:10.1371/journal.pone.0156620)
Supplement: S4 Table — (PDF) [file pone.0156620.s008.pdf]

S4 Table: Effect of snow manipulation on main shoot winter survival and growth of spruce and pine seedlings on 2014 growing season.

Date	Treatment	Dead shoot (%)		Shoot length (cm)	
		Spruce	Pine	Spruce	Pine
6 Jun	AMB	1.0 ± 1.0	5.0 ± 2.2		4.52 ± 0.23
	IE	7.0 ± 2.1	17.1 ± 5.8		3.69 ± 0.37
	COMP	7.0 ± 3.0	13.6 ± 3.1		4.02 ± 0.38
	NoSNOW	10.0 ± 2.1	13.0 ± 4.0		3.93 ± 0.44
11 Jul	AMB	3.0 ± 2.1	5.0 ± 2.2	7.04 ± 0.46	9.40 ± 0.45
	IE	12.0 ± 4.2	19.1 ± 5.6	5.74 ± 0.44	8.41 ± 0.75
	COMP	7.0 ± 3.0	13.6 ± 3.1	6.52 ± 0.42	9.01 ± 0.77
	NoSNOW	11.0 ± 2.3	16.0 ± 4.3	6.36 ± 0.34	9.22 ± 0.97
30 Sep	AMB	3.0 ± 2.1	5.0 ± 2.2	7.06 ± 0.46	9.79 ± 0.46
	IE	12.0 ± 4.2	23.2 ± 5.2	5.76 ± 0.44	8.20 ± 0.78
	COMP	8.0 ± 3.3	13.6 ± 3.1	6.23 ± 0.37	9.04 ± 0.69
	NoSNOW	11.0 ± 2.3	18.0 ± 4.7	6.43 ± 0.34	8.96 ± 0.91
