# Supplementary material for: The Snow Must Go On: Ground Ice Encasement, Snow Compaction and Absence of Snow Differently Cause Soil Hypoxia, CO2 Accumulation and Tree Seedling Damage in Boreal Forest
Source: PLoS One. 2016 Jun 2;11(6):e0156620. doi: 10.1371/journal.pone.0156620 (PMC4890806; doi:10.1371/journal.pone.0156620)
Supplement: S5 Table — (PDF) [file pone.0156620.s009.pdf]

S5 Table. Statistical significance tests (linear mixed model for treatment, date and their interaction) for proportion of dead main shoots and seedling length.

	**Dead shoot (%)**						**Shoot length (cm)**					
	Spruce			Pine			Spruce			Pine		
	df	F	Sig	df	F	Sig	df	F	Sig	df	F	Sig
Treatment	3/28.2	2.27	0.102	3/27.4	2.76	0.061	3/27	3.68	0.024	3/29.6	0.652	0.588
Date	2/72.5	5.03	0.009	2/71.9	3.67	0.030	1/36	0.37	0.547	2/36	154.5	<0.001
Treatment*Date	6/72.5	1.48	0.197	6/71.9	1.45	0.217	3/36	1.2	0.324	6/36	1.62	0.171
